# Remote Identification of APOE-ε4 Carrier Status in Cognitively Normal Adults via Speech Acoustics: Cross-Sectional Observational Study

**DOI:** 10.2196/89830

**Published:** 2026-10-08

**Authors:** Maryam Tavakoli, Mehrdad Dadgostar, Jordan R Green, David H Salat, Steven E Arnold, Kathryn P Connaghan, Brian Richburg, Nelson V Barnett, Mariam Tkeshelashvili, Marziye Eshghi

**Affiliations:** 1 Mass General Brigham University of Health Professions Charlestown, MA United States; 2 Athinoula A. Martinos Center for Biomedical Imaging Massachusetts General Hospital Harvard Medical School Charlestown, MA United States; 3 Speech and Hearing Bioscience and Technology Harvard University Boston, MA United States; 4 Department of Radiology Massachusetts General Hospital Harvard Medical School Boston, MA United States; 5 Neuroimaging Research for Veterans Center VA Boston Healthcare System Jamaica Plain, MA United States; 6 Department of Neurology Massachusetts General Hospital Harvard Medical School Boston, MA United States

**Keywords:** APOE-ε4 genotype, genetic risk factor, Alzheimer disease, speech biomarkers, speech acoustics, eGeMAPS, extended Geneva Minimalistic Acoustic Parameter Set, apolipoprotein E ε4

## Abstract

**Background:**

Apolipoprotein E ε4 (APOE-ε4), the strongest genetic risk factor for late-onset Alzheimer disease (AD), is associated with early neuromotor vulnerability that may precede measurable cognitive decline. Because speech integrates fine neuromotor processes, acoustic analysis could offer a sensitive, noninvasive marker of preclinical effects.

**Objective:**

This study aims to determine if speech acoustics distinguish cognitively normal APOE-ε4 carriers from noncarriers, and to assess which speech tasks provide optimal classification performance.

**Methods:**

In this cross-sectional observational study, 80 cognitively normal adults aged 41 to 89 years (22 APOE-ε4 carriers and 58 noncarriers) completed sustained phonation, oral diadochokinetic syllable repetition, passage reading, and spontaneous-speech tasks. Speech samples were collected remotely using participants’ personal devices and processed to extract 88 acoustic features from the extended Geneva Minimalistic Acoustic Parameter Set. Task-specific random forest classifiers were developed to distinguish APOE-ε4 carriers from noncarriers. A genetic algorithm was used to select informative features, and model performance was evaluated using leave-one-participant-out cross-validation. Performance metrics included balanced accuracy, accuracy, precision, sensitivity, specificity, *F*_1_-score, and receiver operating characteristic area under the curve (ROC-AUC).

**Results:**

Spontaneous speech produced the strongest classification performance, with balanced accuracy of 0.84, accuracy of 0.89, sensitivity of 0.73, specificity of 0.95, *F*_1_-score of 0.78, and ROC-AUC of 0.75. Performance was lower for sustained phonation (*F*_1_-score=0.69), diadochokinetic syllable repetition (*F*_1_-score =0.70 for /ba/ and 0.64 for /pa/), and passage reading (*F*_1_-score=0.61). Combining recordings across tasks reduced performance (*F*_1_-score=0.53), indicating that task-specific acoustic patterns were more informative than pooled data.

**Conclusions:**

Automated analysis of task-specific acoustic speech features, particularly spontaneous speech, internally distinguished cognitively normal APOE-ε4 carriers from noncarriers. These findings support further validation of speech acoustics as a low-burden digital biomarker of preclinical AD risk and suggest that spontaneous-speech tasks may be especially well suited for future remote and longitudinal assessment.

## Introduction

Alzheimer disease (AD), the leading cause of dementia worldwide, has a prolonged preclinical phase during which pathophysiological changes accumulate in the absence of cognitive symptoms [1]. Because neurodegeneration begins years before symptoms emerge, delaying intervention until clinical decline is apparent sharply limits the effectiveness of available treatments and trials. Detecting individuals at this asymptomatic stage remains a major clinical challenge, underscoring the need for scalable, noninvasive biomarkers that can identify at-risk individuals prior to symptomatic onset. Current biomarker approaches such as cerebrospinal fluid analysis and positron emission tomography (PET) imaging, while informative, are invasive, costly, and not scalable for routine screening. Accordingly, there is a critical need for low-burden biomarkers that can be repeatedly acquired outside specialized clinical settings and deployed at population scale.

Remote digital phenotyping offers a promising approach to this challenge by enabling frequent, real-world measurement through widely available personal devices. Within this framework, speech represents a high-information, task-anchored signal that complements passive measures of mobility, sleep, and device interaction and may serve as a “digital vital sign” of neuromotor and cognitive health. Brief speech samples can be collected through smartphones, tablets, or computers without specialized hardware, enabling low-burden, repeated assessment in naturalistic settings. These advantages make speech-based digital biomarkers well suited for decentralized research, longitudinal monitoring of subtle changes in brain health, and potentially scalable risk screening.

The apolipoprotein E ε4 (APOE-ε4) allele is the strongest genetic risk factor for late-onset AD [2] and has been linked to an accelerated rate of cognitive and motor decline in aging populations [3-5]. While the association between APOE-ε4 and memory decline is well established [6,7], emerging evidence suggests broader effects on cognition, including impairments in language function and executive control, in older adults [8-10]. Longitudinal studies indicate that ε4 carriers experience more rapid deterioration in verbal fluency and complex cognitive tasks compared to noncarriers [10]. In parallel, motor dysfunction associated with APOE-ε4 has been observed in both gross and fine motor control, including speech motor function [4,11-13].

Speech and language changes are increasingly recognized as early markers of AD pathology [14-16]. As speech integrates motor planning, executive control, language processing, and memory, it provides a multidomain indicator of brain health and an ecologically valid diagnostic window. Individuals with mild cognitive impairment (MCI) and AD frequently exhibit slowed speech tempo, increased pausing, and reduced semantic complexity [15,17-19]. Recent machine learning studies have leveraged these features to discriminate MCI and AD, achieving accuracies up to 88% in diverse cohorts [20,21]. However, most prior research has focused on language- and cognition-based markers. Because these markers reflect downstream cognitive dysfunction, they often detect pathology only after cognitive symptoms are visible. In contrast, acoustic markers of speech motor control, including prosodic, temporal, spectral, and voice-quality measures, may capture subtle neuromotor changes that emerge before overt cognitive decline and are particularly amenable to remote acquisition. Nonetheless, research specifically targeting motor-based speech changes in cognitively normal but genetically at-risk groups, such as APOE-ε4 carriers, remains very limited.

Emerging evidence shows that motor function declines more rapidly in individuals with the APOE-ε4 allele than in noncarriers [4,22]. This includes losses in muscle strength, gait stability, and coordination, making ε4 carriers an important group for studying neuromotor vulnerability before cognitive symptoms arise. Speech provides a particularly sensitive window into these changes as ε4 carriers exhibit abnormal orofacial muscle recruitment during speech, with increased amplitude, frequency, and synchrony of motor unit activation [13]. These neuromuscular differences can predict ε4 carrier status with area under the curve (AUC) up to 0.90, outperforming cognitive tests [13]. In addition, speech kinematic features including altered lip movement duration, speed, and range can differentiate cognitively normal ε4 carriers from noncarriers, capturing subtle abnormalities in motor control that may signal elevated AD risk [12]. Machine learning models trained on these kinematic features achieved up to 87.5% accuracy for APOE-ε4 classification, further supporting the utility of speech as a high-yield early biomarker [12].

Because speech sounds are produced through precise movements of the lips, tongue, and jaw, even small disruptions in neuromotor control are expected to propagate to measurable changes in acoustic features. While kinematic measures capture details of articulatory movement like the range and timing of lip and jaw motions, acoustic features reflect how these movements are realized in the speech signal, including qualities such as voice and timing. Importantly, kinematic data acquisition typically requires specialized equipment, controlled environments, and technical expertise for data capture and analysis, limiting its feasibility for broad implementation. In contrast, acoustic data can be collected easily and noninvasively using mobile or telehealth platforms, making it highly suitable for large-scale and at-home monitoring. Acoustic features can be extracted automatically from remotely collected recordings on participants’ personal devices, reducing travel, staffing, and equipment demands while supporting repeated sampling in naturalistic settings. Positioning acoustic speech analysis within a device-agnostic, smartphone- and telehealth-based workflow thus creates a practical pathway from laboratory-based speech paradigms to remote, real-world digital phenotyping pipelines for preclinical AD risk stratification.

Advances in automated acoustic analysis and machine learning now enable objective and scalable evaluation of speech patterns, opening new avenues for remote risk screening in preclinical populations [23]. Within this digital phenotyping framework, this study has two primary objectives: (1) to develop and internally validate an automated speech analytics pipeline that classifies cognitively normal APOE-ε4 carriers and noncarriers using acoustic features from the extended Geneva Minimalistic Acoustic Parameter Set (eGeMAPS) [24], and (2) to identify the speech tasks that maximize classification accuracy and are most feasible for brief, repeated remote assessment.

Because speech tasks impose distinct cognitive, linguistic, and motor demands, they elicit different acoustic profiles. Pooling recordings across tasks may therefore introduce heterogeneity that obscures risk-related patterns. Task selection is particularly important for remote monitoring, where assessments must be brief, understandable, low burden, robust to naturalistic recording conditions, and sufficiently informative for repeated measurement. Evaluating tasks separately may therefore identify more sensitive and practical protocols than an indiscriminate “all-in” approach.

Accordingly, this study aimed to develop and internally validate a task-specific acoustic-speech analytics pipeline for distinguishing cognitively normal APOE-ε4 carriers from noncarriers using remotely collected samples. We evaluated sustained phonation, oral diadochokinetic syllable repetition, passage reading, and spontaneous speech, hypothesizing that spontaneous speech would yield the strongest classification performance because it concurrently engages linguistic, executive, memory, and speech-motor processes. To our knowledge, this is among the first studies to classify APOE-ε4 carrier status in cognitively normal adults using eGeMAPS-derived features from remotely collected speech. By identifying informative tasks, this work may guide the development of brief remote-speech protocols for longitudinal monitoring and preclinical risk stratification.

## Methods

### Participant Population

A total of 80 cognitively normal participants (49 females and 31 males; age range: 41-89 years; mean age 70.5, SD 9.78 years) were enrolled in this study. Participants were divided into 2 groups based on their APOE genotype: APOE-ε4 carriers (n=22, 8 males and 14 females) and APOE-ε4 noncarriers (n=58, 23 males and 35 females). The ε4 carrier group included individuals with ε3/ε4 heterozygous and ε4/ε4 homozygous genotypes, while the ε4 noncarrier group consisted of individuals with the ε3/ε3 homozygous genotype. The groups were comparable with respect to years of education (mean 17.76, SD 1.49 years for ε4 noncarriers and mean 17.55, SD 0.74 years for ε4 carriers; *P*=.11) and age (*P*=.54), as well as sex distribution, global cognitive performance, and family history of AD (all *P*>.05). APOE genotyping was conducted using blood samples, and all participants were blind to their respective genotypes. Demographic and clinical characteristics were collected for all participants and are summarized in Table 1.

Cognitive normality was established using available clinical and neuropsychological data. Participants with an available Clinical Dementia Rating (CDR) assessment were classified as cognitively normal if they had a global CDR score of 0 (n=13). For those without a CDR assessment, cognitive normality was defined as neuropsychological performance within normal limits according to Bondi criteria (n=67). Participants with MCI or dementia were excluded. Additional inclusion criteria were (1) capacity to provide electronic informed consent and follow task-specific instructions; (2) native proficiency in American English; (3) no history of speech, language, hearing, psychiatric, or neurological disorders; and (4) no current use of psychoactive medications that could affect study outcomes.

**Table 1:** Demographic and clinical characteristics of cognitively normal participants stratified by APOE-ε4 carrier status.

Variables	All participants (n=80)	Apolipoprotein E -ɛ4 noncarriers (n=58)	Apolipoprotein E -ɛ4 carriers (n=22)	*P* value
Female, n (%)	49 (61.25)	35 (60.34)	14 (63.64)	.78^a^
Family history of AD^b^, n(%)	38 (47.50)	26 (44.83)	12 (54.55)	.43^a^
Age (years), mean (SD)	70.50 (9.78)	70.85 (10.62)	69.59 (7.24)	.54^c^
Total years of education, mean (SD)	17.65 (1.81)	17.76 (1.49)	17.55 (0.74)	.11
MMSE^d^, mean (SD)	29.44 (0.71)	29.40 (0.70)	29.55 (0.74)	.42
MoCA^e^, mean (SD)	27.23 (2.83)	26.88 (3.14)	28.14 (1.49)	.13

^a^*P* values calculated by chi-square test.

^b^AD: Alzheimer disease.

^c^*P* values calculated by *t* test.

^d^MMSE: Mini-Mental State Examination.

^e^MoCA: Montreal Cognitive Assessment.

### Remote Audio Recording

The Lexis Audio Editor app (Pamsys) was installed on participants’ personal devices to facilitate remote speech recording. Participants were instructed to complete recordings in a quiet environment and to maintain a consistent distance between their mouth and the device’s microphone throughout each task. Before recording, participants were instructed to use their typical speaking voice at a comfortable loudness and rate and to minimize extraneous sounds, such as coughing, handling noise, or background conversation. Audio files were recorded at a sampling rate of 44.1 kHz and saved as uncompressed 16-bit WAV files. Recordings were reviewed after collection for signal quality, background noise, clipping, incomplete task completion, and other artifacts. Recordings that did not meet prespecified quality control criteria were excluded or re-collected when feasible.

### Experimental Tasks

Participants completed speech tasks varying in duration and motor-cognitive demands, including sustained vowel phonation, oral diadochokinetic syllable repetition, passage reading, and spontaneous speech. After exclusion of recordings that did not meet prespecified quality control criteria, including excessive ambient noise, poor audio quality, or incomplete responses, the number of valid recordings was 76 for sustained vowel phonation, 78 for diadochokinetic /ba/, 78 for diadochokinetic /pa/, 79 for passage reading, and 79 for spontaneous speech. Detailed task procedures are provided in Multimedia Appendix 1. For each participant, all available spontaneous-speech recordings were concatenated into a single participant-level spontaneous-speech sample before acoustic-feature extraction and classification. This approach maximized the duration and representativeness of spontaneous speech while ensuring that each participant contributed one observation to the task-specific spontaneous-speech model.

### Acoustic Feature Extraction

Digitized audio samples were postprocessed in Audacity (version 3.7.1, Audacity Team) to remove extraneous silences and nonspeech segments. Each recording was manually trimmed to isolate the target speech tasks prior to analysis. Acoustic features were extracted using the open-source openSMILE toolkit (version 2.5.1; audEERING GmbH) with the eGeMAPS configuration [24]. This standardized configuration provides 88 acoustic features per sample, designed to capture central aspects of speech production, including frequency-, energy and amplitude-, and spectral-related characteristics.

The 88 eGeMAPS features are derived from 18 low-level descriptors (LLDs), which encompass frequency measures (pitch, jitter, and formants), energy and amplitude parameters (shimmer, loudness, and harmonics-to-noise ratio [HNR]), and spectral descriptors (mel-frequency cepstral coefficients [MFCCs], spectral flux, alpha ratio, and Hammarberg index). These LLDs are summarized using functionals such as arithmetic mean and coefficient of variation, generating 36 initial parameters. Additional functionals (eg, percentiles, mean, and SD of slopes) applied to pitch and loudness yield 20 further features. The extended set incorporates 7 additional LLDs, primarily cepstral and dynamic parameters, alongside their corresponding functionals and aggregated statistics for voiced and unvoiced regions. Together, these yield 88 features per utterance. The eGeMAPS framework represents an expanded version of the original GeMAPS parameter set, which is widely adopted for standardized voice analysis and has been successfully applied to quantify subtle alterations in speech associated with neurodegenerative and psychiatric disorders [24-27]. The resulting task-specific eGeMAPS feature vectors were used as input to supervised machine-learning models to classify APOE-ε4 carrier status among cognitively normal participants. Genetic algorithm (GA) feature selection and classifier performance were evaluated using leave-one-participant-out cross-validation (LOPOCV), as described below.

### Classifier and Configuration

Given the modest sample size, high-dimensional feature space, and substantial interfeature correlation, we used a random forest classifier with prespecified, conservatively constrained hyperparameters. Models were implemented in Python 3.11 (Python Software Foundation) using the scikit-learn library (version 1.7.1) [28]. Hyperparameters were specified a priori and were not optimized using study outcomes.

Random forest is an ensemble learning method that constructs multiple decision trees using bootstrap samples and random subsets of candidate features at each split, which can help reduce model variance and limit the influence of highly correlated predictors. Specifically, by setting the max_features hyper-parameter to “sqrt,” we restricted the number of features considered at each split to approximately the square root of the total number of features, thereby reducing the influence of correlated variables. The number of estimators was set to 500 to ensure more stable predictions. Trees were constrained with min_samples_split=10, min_samples_leaf=4, and max_depth=4 to prevent overfitting, as excessively deep trees tend to memorize noise and outliers rather than capture generalizable patterns. We also used bootstrapping to enhance diversity among trees and reduce the variance of the resulting ensemble. The class weight hyper-parameter was set to “balanced_subsample” to account for the class imbalance in our data.

### Feature Selection With GA

To enhance classification performance, GA was used to select an informative subset of features from the full eGeMAPS feature set. Feature selection was performed separately for each speech task, resulting in task-specific feature subsets. In the GA, each candidate solution represented a binary feature-selection vector in which each element indicated whether a given eGeMAPS feature was included or excluded. The GA iteratively evolved candidate feature subsets through selection, crossover, and mutation to maximize classification performance [29-31].

The GA population size was set to 100, and the maximum number of generations was set to 150. Early stopping was applied when no improvement in the best fitness score was observed for 30 consecutive generations (20% of the maximum number of generations). The crossover probability was set to 0.80, and the mutation probability was set to 0.20. These settings were selected to balance exploration of the feature-subset search space with convergence on high-performing solutions.

Each candidate feature subset was evaluated using the prespecified random forest classifier and LOPOCV. The mean LOPOCV *F*_1_-score served as the GA fitness function. The *F*_1_-score, defined as the harmonic mean of precision and recall, was selected because it accounts for the relative balance of false-positive and false-negative classifications and is more informative than overall accuracy when class sizes are unequal. Elitism was used to retain the globally best-performing feature subset across generations. At termination, the subset with the highest LOPOCV *F*_1_-score was retained for each task-specific analysis.

### Evaluation Framework

Given the limited sample size, GA-based feature selection was performed using all available data. The performance of each candidate feature subset during GA optimization was assessed using LOPOCV across the full dataset. Because the classifier hyperparameters were specified a priori rather than optimized using the data, no separate validation set was required for hyperparameter selection. Therefore, the performance of the classifier on the final GA-selected feature subset, assessed using the same LOPOCV procedure, represents internal cross-validation performance. Figure 1 outlines the workflow from remote audio recording and acoustic-feature extraction through feature selection, model training, and LOPOCV-based performance evaluation.

In addition to the *F*_1_-score used as the GA fitness metric, we calculated complementary metrics to comprehensively evaluate classification performance. Balanced accuracy, defined as the mean of class-specific recall values, was emphasized because it is less sensitive to class imbalance than standard accuracy. Precision reflects the proportion of predicted carriers who were true carriers; sensitivity reflects the proportion of true carriers correctly identified; and specificity reflects the proportion of noncarriers correctly identified. Finally, the receiver operating characteristic area under the curve (ROC-AUC) quantified the model’s ability to rank carriers above noncarriers across classification thresholds.

**Figure 1:**
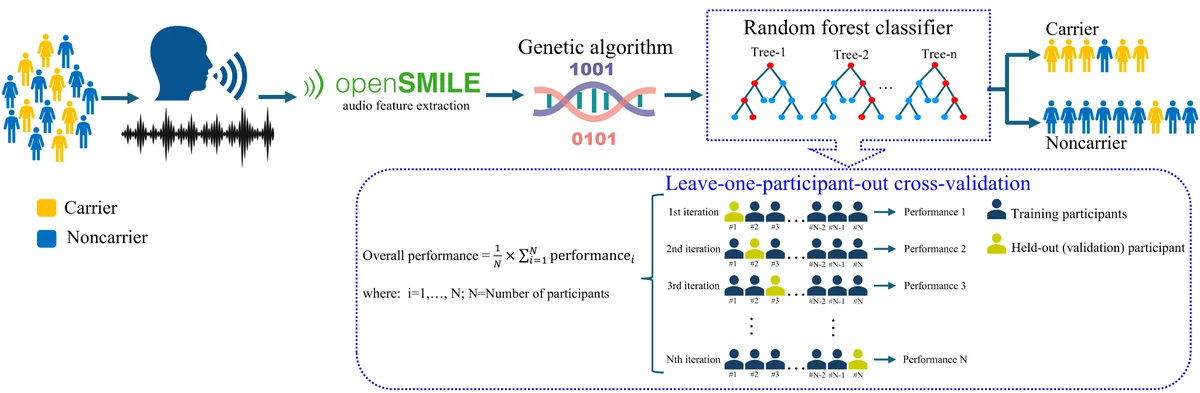
Workflow for remote speech acquisition, audio preprocessing, extended Geneva Minimalistic Acoustic Parameter Set acoustic-feature extraction, task-specific genetic algorithm–based feature selection, random forest classification, and leave-one-participant-out cross-validation–based internal performance evaluation.

### Ethical Considerations

The study was approved by the Mass General Brigham (MGB) Institutional Review Board (IRB protocol number 2025P000136). All procedures were conducted in accordance with MGB IRB guidelines and regulations. Electronic informed consent was obtained from all participants before study participation. This study adheres to the TRIPOD-AI (Transparent Reporting of a multivariable prediction model for Individual Prognosis or Diagnosis-Artificial Intelligence) reporting guidelines. The completed TRIPOD-AI checklist is provided in Multimedia Appendix 2. Before participating in testing, administrators thoroughly explained the experimental tasks and procedures, addressing any questions from participants. Relevant consent forms were provided, and each form was reviewed point by point with potential participants by a research staff member prior to obtaining their signature. Participants were explicitly informed of their right to withdraw from the study at any time during the experiment without any consequences, and all participants provided written informed consent prior to participation.

## Results

### Participant Characteristics

A total of 80 cognitively normal adults were included in the analysis, comprising 22 APOE-ε4 carriers and 58 noncarriers. The groups did not differ significantly in age, years of education, sex distribution, family history of AD, or global cognitive performance as assessed by the Mini-Mental State Examination (MMSE) and Montreal Cognitive Assessment (MoCA; all *P*>.05; Table 1). The absence of group differences on these measured demographic and clinical variables provided a comparable basis for subsequent acoustic classification analyses.

### Task-Specific Classification Performance

GA feature selection was performed separately for each speech task, yielding a distinct task-specific feature subset. The random forest classifier was then trained and evaluated using LOPOCV with the corresponding task-specific GA-selected feature subset. Classification performance across evaluation metrics is presented in Figure 2 and summarized in Table 2.

Spontaneous speech yielded the strongest task-specific classification performance for distinguishing APOE-ε4 carriers from noncarriers. The spontaneous-speech model achieved a balanced accuracy of 0.84, overall accuracy of 0.89, precision of 0.84, sensitivity of 0.73, specificity of 0.95, *F*_1_-score of 0.78, and ROC-AUC of 0.75. The *F*_1_-score for spontaneous speech was higher than those obtained for sustained /a/ phonation (0.69), diadochokinetic /ba/ (0.70), diadochokinetic /pa/ (0.64), and passage reading (0.61). Among structured tasks, diadochokinetic /ba/ achieved the highest balanced accuracy (0.78) and ROC-AUC (0.78), whereas sustained /a/ demonstrated the highest precision (0.92) and specificity (0.98). Passage reading yielded the lowest task-specific *F*_1_-score (0.61) and ROC-AUC (0.66). Across task-specific models, sensitivity ranged from 0.55 for sustained /a/ to 0.73 for spontaneous speech, while specificity ranged from 0.84 for passage reading to 0.98 for sustained /a/.

**Figure 2:**
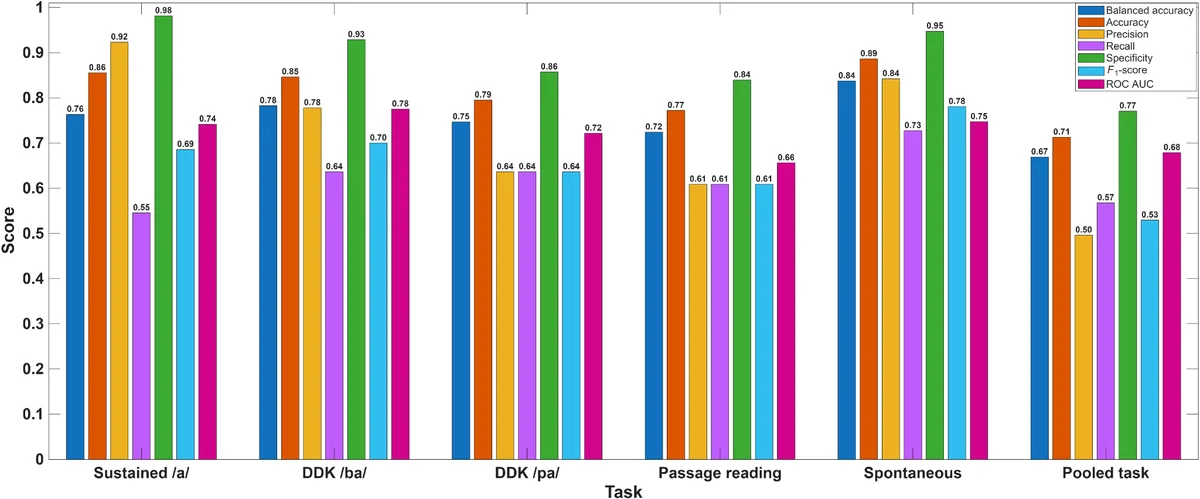
Performance of the random forest classifier across tasks with genetic algorithm-selected features measured using various evaluation metrics. DDK: diadochokinetic; sustained /a/: sustained phonation of the vowel /a/.

**Table 2:** Internal cross-validation performance of task-specific and pooled task random forest classifiers using genetic algorithm-selected extended Geneva Minimalistic Acoustic Parameter Set acoustic features.

Task	Balanced accuracy	Accuracy	Precision	Sensitivity	Specificity	*F*_1_- score	ROC-AUC^a^
Sustained /a/	0.76	0.86	0.92	0.55	0.98	0.69	0.74
DDK^b^ /ba/	0.78	0.85	0.78	0.64	0.93	0.70	0.78
DDK /pa/	0.75	0.79	0.64	0.64	0.86	0.64	0.72
Passage reading	0.72	0.77	0.61	0.61	0.84	0.61	0.66
Spontaneous	0.84	0.89	0.84	0.73	0.95	0.78	0.75
Pooled task	0.67	0.71	0.50	0.57	0.77	0.53	0.68

^a^ROC-AUC: receiver operating characteristic area under the curve.

^b^DDK: diadochokinetic.

### Pooled-Task Classification Performance

In a separate pooled-task analysis, recordings from all speech tasks were aggregated and analyzed using a single GA-selected feature subset. Because each participant contributed multiple recordings to the pooled dataset, LOPOCV was applied at the participant level, such that recordings from a given participant were assigned exclusively to either the training or test set within each fold. This procedure prevented participant-level leakage between training and test data. The pooled-task model showed lower internal cross-validation performance than every task-specific model. It achieved a balanced accuracy of 0.67, accuracy of 0.71, precision of 0.50, sensitivity of 0.57, specificity of 0.77, *F*_1_-score of 0.53, and ROC-AUC of 0.68. The pooled-task *F*_1_-score was lower than the lowest task-specific *F*_1_-score, observed for passage reading (*F*_1_-score=0.61).

### Sex Effect

To examine potential demographic influences on classification performance, sex was included as a categorical predictor alongside the eGeMAPS acoustic features. Including sex did not improve model performance across the evaluated speech tasks. Age was also made available to the GA during feature selection but was not retained in the final feature subset. Given the limited sample size, particularly the number of APOE-ε4 carriers, sex-stratified analyses were underpowered and were not conducted.

### Feature Contributions to APOE-ε4 Classification

Across tasks, GA-selected feature subsets included acoustic measures from fundamental frequency, formant, spectral, MFCC, loudness, voice quality, and segment duration domains (Figure 3). The number and identity of selected features differed across tasks. Spectral features were selected across all task conditions. MFCCs were selected for sustained /a/, DDK /ba/, passage reading, spontaneous speech, and the pooled-task model, but not for diadochokinetic /pa/. F0 and formant-related features were selected for the spontaneous-speech model. Loudness features were selected for sustained /a/, diadochokinetic /ba/, diadochokinetic /pa/, and passage-reading models. Voice quality and segment duration features were selected across multiple tasks.

**Figure 3:**
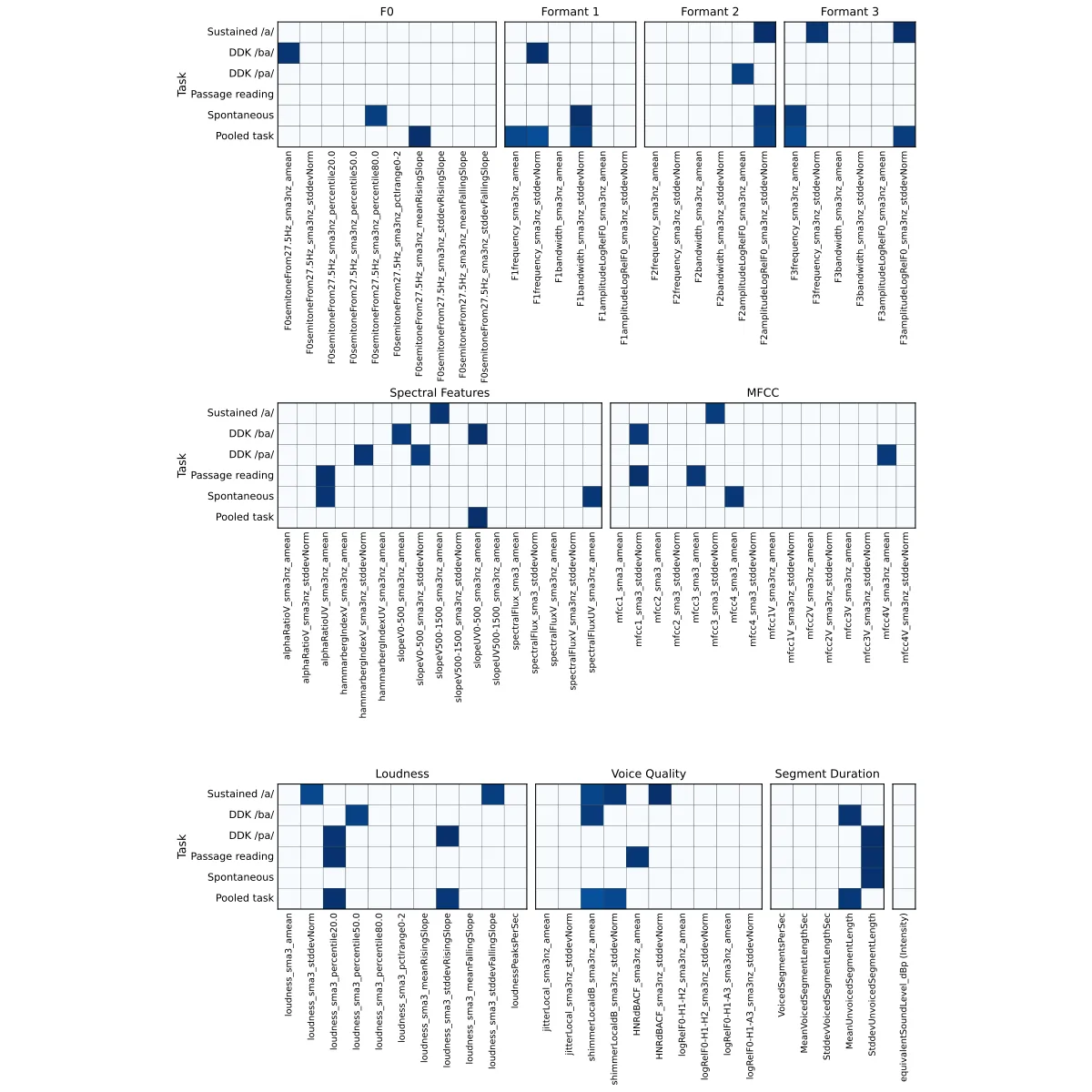
Task-specific extended Geneva Minimalistic Acoustic Parameter Set acoustic features selected by the genetic algorithm for random forest classification of apolipoprotein E ε4 carrier status. Blue cells indicate features retained in the genetic algorithm-selected subset for each speech-task model. The pooled-task condition includes recordings aggregated across all speech tasks. DDK: diadochokinetic; MFCC: mel-frequency cepstral coefficients.

### Shapley Additive Explanations Feature-Attribution Results

A Shapley additive explanations (SHAP) analysis was conducted for the GA-selected features in each task-specific and pooled-task random forest model (Figure 4). Features within each panel are ordered by decreasing mean absolute SHAP value. Point color denotes feature value, with blue indicating lower values and red indicating higher values. Positive SHAP values indicate a higher model-predicted probability of APOE-ε4 carrier status. Because the spontaneous-speech model yielded the highest *F*_1_-score among task-specific models, the following results focus on that model. The highest-ranked spontaneous-speech features were as follows:

- F1bandwidth_sma3nz_stddevNorm
- StddevUnvoicedSegmentLength
- F2amplitudeLogRelF0_sma3nz_stddevNorm
- alphaRatioUV_sma3nz_amean
- spectralFluxUV_sma3nz_amean
- mfcc4_sma3_amean

Higher values of *F1bandwidth_sma3nz_stddevNorm* and lower values of *StddevUnvoicedSegmentLength* were associated with negative SHAP values. Higher values of F2amplitudeLogRelF0_sma3nz_stddevNorm and *mfcc4_sma3_amean* were associated with positive SHAP values. SHAP results for the remaining speech tasks and the pooled-task model are shown in Figure 4.

**Figure 4:**
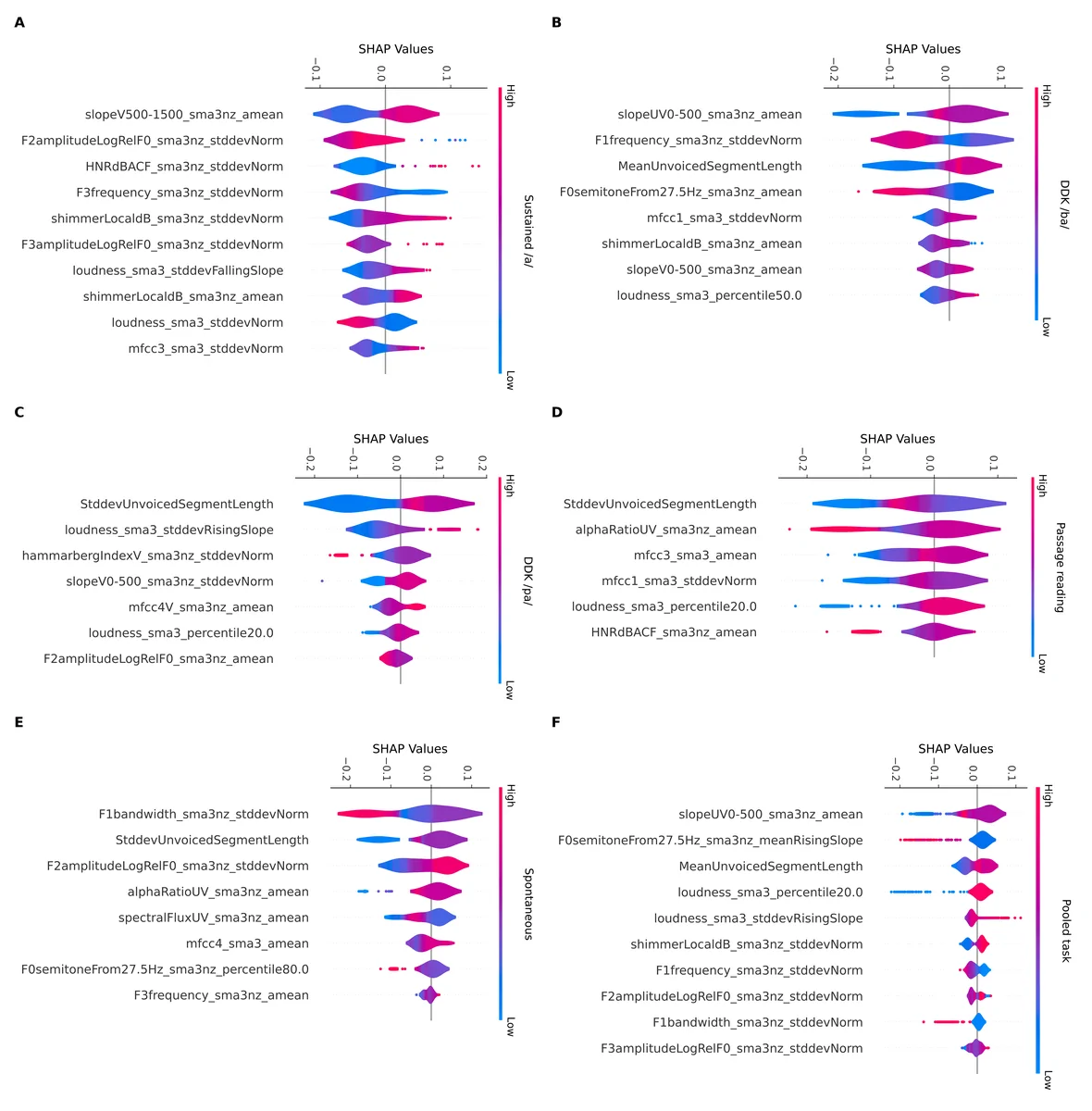
Shapley additive explanations (SHAP) summary plots for genetic algorithm–selected extended Geneva Minimalistic Acoustic Parameter Set acoustic features in random forest models classifying apolipoprotein E ε4 carrier status. Panels display results for (A) sustained /a/ phonation, (B) diadochokinetic /ba/, (C) diadochokinetic /pa/, (D) passage reading, (E) spontaneous speech, and (F) the pooled-task condition. Features within each panel are ordered by decreasing mean absolute SHAP value. Each point represents an observation; color indicates the feature value, from low (blue) to high (red). Positive SHAP values indicate a higher model-predicted probability of apolipoprotein E ε4 carrier status, whereas negative SHAP values indicate a lower probability. DDK: diadochokinetic; sustained /a/: sustained phonation of the vowel /a/.

## Discussion

### Principal Findings

This study found that automated, task-specific acoustic analysis of speech distinguished APOE-ε4 carriers from noncarriers among cognitively normal adults. Across the speech tasks evaluated, spontaneous speech produced the strongest internal cross-validation performance, with an *F*_1_-score of 0.78, balanced accuracy of 0.84, and overall accuracy of 0.89. In contrast, the pooled-task model performed less well than each task-specific model, with an *F*_1_-score of 0.53 compared with 0.61-0.78 for individual tasks. These findings suggest that speech acoustics may contain task-dependent information associated with APOE-ε4 carrier status before clinically apparent cognitive impairment. However, the results should be interpreted as internally validated associations with genetic AD risk, not as evidence that speech acoustics detect biomarker-confirmed preclinical AD, predict future cognitive decline, or establish an individual’s clinical risk.

### Task-Specific Acoustic Profiles

The feature-selection map and SHAP analyses indicated that APOE-ε4 carrier-status classification was supported by multivariate acoustic profiles rather than by a single task-invariant marker. Across tasks, selected features encompassed spectral, cepstral, formant-related, temporal, loudness, and voice quality domains. The specific features retained differed across sustained phonation, diadochokinetic /pa/, diadochokinetic /ba/, passage reading, spontaneous speech, and the pooled-task condition. This task dependence is plausible because the speech paradigms sample partially distinct aspects of speech production. Sustained phonation primarily reflects phonatory control and vocal stability; diadochokinetic tasks impose demands on rapid, repetitive articulatory sequencing; passage reading constrains lexical and syntactic content while retaining prosodic and articulatory demands; and spontaneous speech additionally requires online linguistic formulation, lexical retrieval, discourse organization, working-memory engagement, executive control, and continuous speech-motor planning.

Spectral features were selected across all task conditions, suggesting that spectral information contributed relatively consistently to carrier-status classification in this sample. These features characterize the distribution of acoustic energy across frequencies and may reflect a combination of laryngeal-source characteristics, vocal-tract filtering, articulatory configuration, and temporal coordination. MFCCs, which summarize broad properties of the spectral envelope, were selected for sustained /a/, diadochokinetic /ba/, passage reading, spontaneous speech, and the pooled-task condition, but not for diadochokinetic /pa/. Their recurrence across several, but not all, conditions further indicates that the informativeness of specific acoustic domains depends on the speech task.

The models also retained HNR, shimmer, loudness, spectral slope, and segment duration measures, which capture complementary aspects of periodicity and noise, amplitude-related characteristics, spectral-envelope shape, and speech timing. Prior studies of MCI and AD have similarly examined temporal measures such as pausing, speech rate, speech time, and phonation time, as well as spectral and voice-related measures including MFCCs, pitch, jitter, shimmer, and voice breaks [32-34]. However, the selected features and the direction and magnitude of their SHAP attributions varied across tasks. They should therefore be interpreted as properties of the fitted models in this dataset, rather than as evidence of a uniform APOE-ε4 acoustic phenotype or direct measures of neurodegeneration, cognition, or a specific laryngeal, respiratory, articulatory, or neural mechanism.

### Spontaneous Speech

Spontaneous speech yielded the strongest task-specific classification performance and incorporated F0- and formant-related features, spectral measures, MFCCs, and temporal features. This pattern indicates that performance reflected information across multiple acoustic domains rather than a single dominant marker. In SHAP analyses, higher values of *F2amplitudeLogRelF0_sma3nz_stddevNorm* and *mfcc4_sma3_amean* were associated with a higher model-predicted probability of APOE-ε4 carrier status. Conversely, higher values of *F1bandwidth_sma3nz_stddevNorm* and lower values of *StddevUnvoicedSegmentLength* were associated with a lower predicted carrier probability. These directions describe how the fitted model used the features in this sample; they do not establish a stable group-level difference, a biological mechanism, or a stand-alone biomarker.

F0 indexes fundamental frequency and captures aspects of pitch regulation and prosodic control. Formant frequencies and bandwidths are acoustic correlates of vocal tract resonance and articulatory configuration, while MFCCs characterize broad properties of the spectral envelope. Temporal features characterize aspects of speech timing and segmentation. The contribution of these feature families may reflect the broad demands imposed by spontaneous speech. Unlike sustained phonation and DDK tasks, spontaneous speech requires speakers to formulate content online, select and retrieve words, maintain discourse coherence, regulate prosody, coordinate articulation and respiration, and organize the timing of connected speech. This combination of cognitive-linguistic and speech-motor demands may provide more opportunities for subtle acoustic variation associated with APOE-ε4 carrier status to emerge. Because this study did not directly assess lexical retrieval, executive function during speech, articulation, respiratory function, laryngeal function, or other proposed contributors, this explanation remains hypothesis generating.

The feature domains contributing to the spontaneous speech model are broadly consistent with prior eGeMAPS-based research in MCI [35]. García-Gutiérrez et al [35] identified formant bandwidth, spectral, and voiced segment duration measures among the acoustic variables contributing to amyloid status classification in individuals with MCI; their best acoustic model achieved 75% accuracy and an AUC of 0.79. This comparison is descriptive rather than confirmatory. García-Gutiérrez et al [35] evaluated amyloid status in a clinical MCI cohort, whereas this study evaluated APOE-ε4 carrier status in cognitively normal adults. Thus, overlap in feature families does not demonstrate that the speech patterns observed here reflect amyloid pathology.

Prior literature supports the value of connected and spontaneous speech for identifying language, communication, acoustic, and temporal differences associated with MCI and AD [36-38]. Reviews of connected-speech research indicate that picture description and other discourse-level tasks can reveal changes associated with cognitive impairment, but they also emphasize substantial heterogeneity in elicitation methods, participant populations, speech measures, languages, target outcomes, and analytic approaches [36,37]. Automated speech and language research similarly suggests that spontaneous speech can provide information-rich acoustic and linguistic data for modeling cognitive impairment and AD-related outcomes, although reported accuracy varies materially across tasks, cohorts, and validation designs [21,38,39].

Task selection may therefore be as important as the analytic model itself. Clarke et al [40] compared five connected-speech tasks in healthy controls and participants with MCI or mild AD and found that accuracy varied from 0.62 for novel narrative retelling using a wordless picture book to 0.78 for overlearned narrative recall. Overlearned narrative recall also achieved the highest specificity, at 0.82. Similarly, the current finding that the pooled-task model performed worse than task-specific models suggests that heterogeneous recordings should not be assumed to provide additive information. Combining sustained phonation, diadochokinetic, passage-reading, and spontaneous-speech recordings introduces variation in duration, linguistic content, speaking style, task instructions, and cognitive-motor demands that may obscure task-specific acoustic patterns.

### Implications for Digital Phenotyping

These findings support continued investigation of speech acoustics as a low-burden component of digital phenotyping for AD-related risk. Speech can be recorded repeatedly using common personal devices and may enable within-person monitoring of acoustic, temporal, prosodic, and linguistic measures over time. Spontaneous speech is especially relevant for future work because it can be elicited through brief narrative prompts, picture descriptions, or semistructured interviews. However, the stronger performance of spontaneous speech in this dataset does not establish that it is universally superior to structured speech tasks. Its performance should be compared prospectively with other standardized tasks in independent cohorts.

Remote repeated connected-speech assessment has been shown to be feasible and reliable in cognitively unimpaired adults, including participants with and without amyloid-beta pathology [41]. In a 5-day tablet-based protocol repeated 2-3 weeks later, adherence was 91.6%, mean usability was 86.0 (SD 9.9), and averaged speech samples demonstrated high test-retest reliability (intraclass correlation coefficient [ICC] ≥0.75) [41]. These findings support the feasibility of remote speech collection but do not eliminate concerns about measurement variation. Recording device, microphone characteristics, room acoustics, background noise, microphone-to-mouth distance, connectivity, task adherence, response length, and prompt content can influence recording quality and extracted acoustic measures [42-44]. Future protocols should standardize prompts and recording duration, collect device metadata, implement audio quality thresholds, and evaluate model robustness and calibration across common devices and real-world recording environments.

Speech-based measures are most appropriately considered potential complements to established clinical, neuropsychological, imaging, and fluid biomarkers rather than replacements for them. APOE-ε4 is a major genetic risk factor for late-onset AD [45,46], but carrier status does not establish the presence of amyloid or tau pathology, and risk relationships can vary across populations [47,48]. Therefore, the immediate value of speech acoustics may lie in repeated research assessments, characterization of within-person change, cohort enrichment, and multimodal risk modeling. Any eventual clinical use would require evidence that speech features contribute incremental information beyond established demographic, genetic, cognitive, imaging, and fluid-biomarker measures.

### Limitations and Future Directions

Several limitations should guide interpretation. First, the modest sample size, including the number of APOE-ε4 carriers, limits statistical power, the precision of performance estimates, and generalizability. The reported *F*_1_-score and balanced accuracy appropriately provide information beyond overall accuracy, particularly if the carrier and noncarrier groups were imbalanced; nevertheless, confidence intervals should be reported for all key performance metrics where feasible. Sex-stratified analyses were underpowered. Although sex was included as a candidate predictor and did not improve classifier performance in this dataset, that result should not be interpreted as evidence that sex is unrelated to APOE-ε4–associated acoustic patterns. Larger, demographically balanced cohorts are needed to assess the effects of age, sex, education, race and ethnicity, language background, multilingualism, hearing status, vocal health, and regional or social speech variation on both acoustic-feature distributions and model performance. Second, the study did not examine the independent or interactive effects of factors that may influence speech and AD-related risk. These include vascular and cardiometabolic health, medication use, depression and anxiety, sleep disturbance, respiratory disease, neurological comorbidities, tobacco or alcohol exposure, and other conditions affecting voice, communication, or cognition. Future models should include these variables to assess possible confounding, effect modification, and calibration across clinically heterogeneous populations. Third, the cross-sectional design precludes conclusions regarding temporality, prognosis, or disease progression. The observed acoustic patterns may not precede cognitive decline, correspond to amyloid or tau pathology, or change as AD-related risk evolves. Longitudinal studies should combine repeated task-specific speech recordings with cognitive testing, amyloid and tau biomarkers, neuroimaging, and clinically meaningful outcomes. Such studies can determine whether speech features predict cognitive decline, track within-person change, provide prognostic information, or add value to multimodal models. Finally, the reported performance estimates were derived from internal cross-validation and require confirmation in independent external cohorts. Future studies should evaluate the complete feature-selection and model development pipeline in prospectively collected datasets to assess generalizability and reduce the risk of optimistic performance estimates.

### Conclusion

Automated analysis of speech acoustics, particularly from spontaneous speech, distinguished APOE-ε4 carriers from noncarriers among cognitively normal adults under internal cross-validation. These findings suggest that standardized acoustic features combined with machine-learning methods may capture subtle speech differences associated with genetic AD risk before overt cognitive impairment. However, because APOE-ε4 carrier status does not establish biomarker-confirmed preclinical AD, the identified features should not be interpreted as detecting AD pathology or predicting individual clinical outcomes. Longitudinal studies incorporating amyloid and tau biomarkers and cognitive outcomes are needed to determine whether these acoustic patterns track disease progression or predict subsequent decline. Speech-based assessment may ultimately provide a scalable, low-burden complement to established clinical, neuropsychological, imaging, and fluid biomarker measures, particularly for repeated monitoring and multimodal risk assessment. Clinical translation will require prospective external validation in larger, diverse cohorts, evaluation across devices and recording environments, and demonstration of measurement reliability, model calibration, and incremental value beyond established AD risk and biomarker measures.

## Appendix

Multimedia Appendix 1 Description and rationale for sustained /a/ phonation, oral diadochokinetic /ba/ and /pa/ repetition, bamboo passage reading, and spontaneous speech tasks.

Multimedia Appendix 2 TRIPOD+AI checklist.

## Abbreviations

AD: Alzheimer disease
APOE-ε4: apolipoprotein E ε4
CDR: Clinical Dementia Rating
eGeMAPS: extended Geneva Minimalistic Acoustic Parameter Set
GA: genetic algorithm
HNR: harmonics-to-noise ratio
IRB: institutional review board
LOPOCV: leave-one-participant-out cross-validation
LLD: low-level descriptor
MGB: Mass General Brigham
MFCC: mel-frequency cepstral coefficient
MCI: mild cognitive impairment
MMSE: Mini-Mental State Examination
MoCA: Montreal Cognitive Assessment
PET: positron emission tomography
ROC-AUC: receiver operating characteristic area under the curve
SHAP: Shapley additive explanations
TRIPOD-AI: Transparent Reporting of a multivariable prediction model for Individual Prognosis or Diagnosis-Artificial Intelligence

## Data Availability

The raw speech recordings generated during the current study contain personally identifiable information and cannot be shared publicly for privacy and ethical reasons. A deidentified dataset comprising the extracted acoustic features that support the findings of this study is available from the corresponding author upon reasonable request.
