# Supplementary material for: Remote Identification of APOE-ε4 Carrier Status in Cognitively Normal Adults via Speech Acoustics: Cross-Sectional Observational Study
Source: JMIR Form Res. 2026 Oct 8;10:e89830. doi: 10.2196/89830 (PMC13691465; doi:10.2196/89830)
Supplement: Supplementary file 2 [file formative_v10i1e89830_app1.docx]

**Appendix 2**

*Sustained phonation task:* Participants were asked to take a deep breath and prolong the vowel /a/ for as long as possible, at their typical pitch and loudness. The sustained phonation task is widely used in voice assessment and therapy, as it provides critical insights into glottic function (i.e., vocal fold closure) and respiratory-phonatory coordination [1], while offering a controlled context for precise acoustic measurements. This task is particularly valuable for acoustic analysis because it yields a stable and continuous vocal signal, minimizing variability from pitch modulation, loudness changes, and articulatory influences seen in connected speech. By reducing the effects of coarticulation and prosodic variation, sustained phonation enables precise extraction of key acoustic features such as fundamental frequency (F0), jitter, and shimmer. These acoustic parameters are commonly used to assess voice quality and detect vocal pathology. In addition to its diagnostic utility, the simplicity of the task facilitates standardized comparisons across individuals and time points.

*Syllable repetitions in an oral diadochokinetic paradigm:* Oral DDK tasks are clinical tools used to assess oromotor coordination, speed, and precision by measuring rapid syllable repetition. These tasks include Alternating Motion Rate (AMR) tasks which involve the rapid and accurate repetition of a single syllable on a single breath and are commonly used to evaluate execution-level motor speech control. In this study, we used the syllables /ba/ and /pa/, which differ in voicing (i.e., /ba/ being voiced and /pa/ voiceless) while both require bilabial closure. These differences allow us to examine subtle variations in articulatory timing and control. Impaired AMR performance, such as slowed rate or increased variability, can indicate neuromuscular dysfunction, commonly observed in disorders like dysarthria. AMR tasks are also sensitive to subtle changes in articulatory speed and stability across age groups or neurological conditions [2, 3]. While these tasks do not fully capture the complexity of spontaneous speech, they provide a controlled and quantifiable measure of speech motor performance, offering valuable insights into oromotor integrity and efficiency.

*Passage reading:* In this study participants were asked to read the “Bamboo Passage” which is a 60-word paragraph containing voiced consonants (e.g., /b/ and /d/) at word and phrase boundaries to eliminate misidentification of pauses that can occur during voiceless stops. This task provides sensitive measures of motor speech fluency including speaking rate, pause duration, and articulatory precision and has been used in dysarthria research to identify errors in fine-motor speech sounds, with multiple studies showing it can distinguish individuals with early-stage ALS from healthy controls [4, 5]. The passage’s standardized content enables semi-automated, reliable tracking of subtle speech changes for diagnosis, and disease progression and treatment response in clinical trials. In addition to capturing motor deficits, performance on the task reflects cognitive functions such as sustained attention, visual discrimination, processing speed, and linguistic processing (phonology, semantics, syntax). While repeated use may introduce practice effects and its structure may not fully represent spontaneous speech, the Bamboo Passage remains a robust tool for identifying early motor speech changes and developing speech-based biomarkers especially when used alongside naturalistic tasks for a comprehensive assessment.

*Spontaneous speech samples:* Participants were asked to speak for one minute on each of the following prompts: (1) describe what a typical weekend looks like for you, (2) describe your hobbies and activities, and (3) describe a place or country you have visited and particularly enjoyed. These open-ended tasks are designed to tap into natural language production abilities, engaging a broad array of cognitive-linguistic and speech motor processes including lexical retrieval, syntactic construction, articulation, and prosodic modulation. Unlike structured tasks, spontaneous speech offers a more ecologically valid window into real-world communication, making it especially valuable for detecting subtle deficits that may not emerge in constrained contexts. When combined with automated speech analysis, these samples can be systematically evaluated for acoustic and linguistic features, enhancing sensitivity to early cognitive or neuromotor changes, supporting reproducible and scalable analysis, and enabling longitudinal tracking of disease progression or treatment response in clinical populations.

## **References**

1.	Maslan J, Leng X, Rees C, et al (2011) Maximum phonation time in healthy older adults. J Voice 25:709–713. https://doi.org/10.1016/j.jvoice.2010.10.002

2.	Ben-David BM, Icht M (2017) Oral-diadochokinetic rates for Hebrew-speaking healthy ageing population: Non-word versus real-word repetition. Int J Lang Commun Disord 52:301–310. https://doi.org/10.1111/1460-6984.12272

3.	Tafiadis D, Zarokanellou V, Prentza A, et al (2022) Diadochokinetic rates in healthy young and elderly Greek‐speaking adults: The effect of types of stimuli. Int J Lang & Commun Disord 57:1085–1097. https://doi.org/10.1111/1460-6984.12747

4.	Yunusova Y, Green JR, Wang J, et al (2011) A Protocol for Comprehensive Assessment of Bulbar Dysfunction in Amyotrophic Lateral Sclerosis (ALS). J Vis Exp. https://doi.org/10.3791/2422

5.	Simmatis LE, Robin J, Pommée T, et al (2023) Validation of automated pipeline for the assessment of a motor speech disorder in amyotrophic lateral sclerosis (ALS). Digit Heal 9:20552076231219104. https://doi.org/10.1177/20552076231219102
